# Isolation and Functional Analysis of Genes Involved in Polyacylated Anthocyanin Biosynthesis in Blue *Senecio cruentus*

**DOI:** 10.3389/fpls.2021.640746

**Published:** 2021-02-22

**Authors:** Chenfei Lu, Yajun Li, Yumeng Cui, Jiangshan Ren, Fangting Qi, Jiaping Qu, He Huang, Silan Dai

**Affiliations:** Beijing Advanced Innovation Center for Tree Breeding by Molecular Design, Beijing Key Laboratory of Ornamental Plants Germplasm Innovation and Molecular Breeding, National Engineering Research Center for Floriculture, Beijing Laboratory of Urban and Rural Ecological Environment, Key Laboratory of Genetics and Breeding in Forest Trees and Ornamental Plants of Education Ministry, School of Landscape Architecture, Beijing Forestry University, Beijing, China

**Keywords:** blue flower, polyacylated anthocyanins, *Senecio cruentus*, acyltransferase, glucosyltransferase

## Abstract

Polyacylated anthocyanins with multiple glycosyl and aromatic acyl groups tend to make flowers display bright and stable blue colours. However, there are few studies on the isolation and functional characterization of genes involved in the polyacylated anthocyanin biosynthesis mechanism, which limits the molecular breeding of truly blue flowers. *Senecio cruentus* is an important potted ornamental plant, and its blue flowers contain 3′,7-polyacylated delphinidin-type anthocyanins that are not reported in any other plants, suggesting that it harbours abundant gene resources for the molecular breeding of blue flowers. In this study, using high-performance liquid chromatography-tandem mass spectrometry (HPLC-MS/MS) analysis of blue, carmine and white colours of cineraria cultivars “Venezia” (named VeB, VeC, and VeW, respectively), we found that 3′,7-polyacylated anthocyanin, cinerarin, was the main pigment component that determined the blue colour of ray florets of cineraria. Based on the transcriptome sequencing and differential gene expression (DEG) analysis combined with RT- and qRT-PCR, we found two genes encoding uridine diphosphate glycosyltransferase, named *ScUGT1* and *ScUGT4*; two genes encoding acyl-glucoside-dependent glucosyltransferases which belong to glycoside hydrolase family 1 (GH1), named *ScAGGT11* and *ScAGGT12*; one gene encoding serine carboxypeptidase-like acyltransferase *ScSCPL2*; and two MYB transcriptional factor genes *ScMYB2* and *ScMYB4*, that were specifically highly expressed in the ray florets of VeB, which indicated that these genes may be involved in cinerarin biosynthesis. The function of *ScSCPL2* was analysed by virus-induced gene silencing (VIGS) in cineraria leaves combined with HPLC-MS/MS. *ScSCPL2* mainly participated in the 3′ and 7-position acylation of cinerarin. These results will provide new insight into the molecular basis of the polyacylated anthocyanin biosynthesis mechanism in higher plants and are of great significance for blue flower molecular breeding of ornamental plants.

## Introduction

Flower colour is one of the most important traits of ornamental plants. Bright and stable blue flowers are precious and scarce resources in nature, and they are popular among consumers for their dream-like visual experience (Noda, 2018). Blue floral colour primarily arises from anthocyaninss, which are modified by aromatic or fatty acyl groups that are commonly linked to the hydroxy groups of the glycosyl moieties. Anthocyanins modified with multiple aromatic acyl groups are often referred to as polyacylated anthocyanins, and the colour shifts to blue gradually as the number of acyl groups increases (Yoshida et al., 2009; Tanaka and Brugliera, 2013). Meanwhile, polyacylation at the 3′ or 7-position of anthocyanin is more important for stable blue coloration, while the 3- or 5-position appears to provide only a reddish-purple colour (Matsuba et al., 2010; Nishizaki et al., 2013; Yoshida and Negishi, 2013). Although many different polyacylated anthocyanin structures have been identified from different species, isolation and functional studies of genes involved in polyacylation modification are few, which limits the molecular breeding of truly blue flowers.

The anthocyanin biosynthesis pathway in higher plants is one of the most clearly elucidated secondary metabolic pathways, mainly including the metabolic enzymes such as chalcone synthase (CHS), chalcone isomerase (CHI), flavanone 3-hydroxylase (F3H), flavonoid 3′-hydroxylase (F3′H), flavonoid 3′,5′-hydroxylase (F3′5′H), dihydroflavonol 4-reductase (DFR), anthocyanidin synthase (ANS), glycosyltransferase (GT), and acyltransferase (AT) (Liu et al., 2018; Amoanimaa-Dede et al., 2019). Polyacylation modification, which is crucial for blue flower coloration, is catalysed by GTs and ATs in an appropriate order, among which uridine diphosphate-dependent glycosyltransferase family 1 (UGT) and glycoside hydrolase family 1 (GH1) are two widely studied enzyme families known to transfer glycosyl groups (Sasaki and Nakayama, 2015). UGTs can transfer glycosyls from UDP sugars to flavonoids, and their catalytic function can be divided into 4 clades according to phylogenetic analysis. UGTs from Clades I and II catalyse the glycosylation of the 3- and 5-*O*-position of anthocyanins, respectively, which is the key step for the formation of stable anthocyanins and is also the most conventional and common glycosylation modification. UGTs from Clade III catalyse the glycosylation of 3′ or 7-*O*-position of flavonoids, which is involved in the biosynthesis of polyacylated anthocyanins (Nakajima et al., 2001; Yonekura-Sakakibara and Hanada, 2011; Sasaki and Nakayama, 2015). Acyl-glucoside-dependent glucosyltransferases (AGGTs), which belong to glycoside hydrolase family 1 (GH1), catalyse the glycosylation of anthocyanins with acyl-glucoses as glucosyl donors. AGGTs were identified for the first time in carnation and delphinium (named DcAA5GT and DgAA7GT, respectively), which are known to accumulate in vacuoles and contain strict substrate preferences (Luang et al., 2013; Matsuba et al., 2010).

Anthocyanin acyltransferase (AAT) is responsible for catalysing the acylation of anthocyanins after glycosyl connections. Serine carboxypeptidase-like (SCPL)-AATs are a type of enzymes that belong to the α/β hydrolase family. These enzymes have acyltransferase activity of secondary metabolites, involving advanced and even final modification of anthocyanins after being transported into the vacuole. Anthocyanins with multiple aromatic acyl groups (often referred to as polyacylated anthocyanins) are the main products catalysed by acyltransferase (Ciarkowska et al., 2018), and the stacking of aromatic acyl groups makes the anthocyanins appear blue. According to phylogenetic evolution, *SCPL*s can be clustered into three clades, Clade IA, Clade IB, and Clade II. Among them, members of Clade IA generally have acyltransferase activity, and the expression of these genes is specific to tissues and organs, while other *SCPL* genes are widely expressed in different plant tissues (Fraser et al., 2005). At present, *SCPL* genes involved in acylation of anthocyanins and other flavonoids have been identified from *Clitoria ternatea*, *Delphinium grandiflorum*, and *Diospyros kaki* (Noda et al., 2006; Milkowski and Strack, 2010; Akagi et al., 2011; Nishizaki et al., 2013). However, the mechanism for some glycosylation and acylation steps is variable in different species. Thus, clarifying more modification mechanisms and identifying more key genes will be conducive to the blue flower molecular breeding of ornamental plants.

In addition, the transcription of structural genes related to anthocyanin synthesis is mainly regulated by the MYB-bHLH-WD40 (MBW) transcriptional complex, among which MYB transcription factors are the most important. Most studies of MYB found that it could regulate the entire pathway of anthocyanin metabolism. In recent years, many studies have found that different members of the MYB family are likely to carry out specific regulation on different branches of anthocyanin metabolism and biochemical modification (Nakatsuka et al., 2008; Chen et al., 2019). For example, in *Hordeum vulgare* and *Gentiana triflora*, MYB transcription factors can directly regulate the expression of *F3*′*5*′*H* (Nakatsuka et al., 2008; Jia et al., 2020). *PpMYB9* and *PpMYB10.2* in *Prunus persica* had transcriptional activation of the glycosylation genes *PpUGT78A2* and *PpUFGT*, respectively (Hui et al., 2016). However, there are few studies of the transcriptional regulation mechanism of polyacylation modification in plants.

Cineraria (*Senecio cruentus*), which is an important potted flower in Compositae, is widely used in landscapes and has high ornamental and economic value. As the major pigment component in cineraria, Dp3MalG-7d(CafG)-3′CafG (cinerarin) has 3′ and 7-position polyacylation simultaneously (Suzuki et al., 2003; Sun et al., 2010; Sasaki and Nakayama, 2015). The 3′ and 7-position polyacylation of cinerarin is key for the bright blue coloration of cineraria. However, there are few reports of gene isolation, functional verification and metabolic regulation involved in this metabolic process, which limits the breeding of truly blue flowers. In this study, we analysed the pigment composition of blue, carmine and white cineraria cultivars “Venezia” by HPLC-MS/MS and screened out the key genes encoding glycolyltransferase and acyltransferase, as well as transcription factors that may be involved in the polyacylation modification of anthocyanins, using comparative transcriptomics and gene expression analysis. While the flower colour shifts to blue gradually as the number of acyl groups catalysed by acyltransferase increases (Yoshida et al., 2009; Tanaka and Brugliera, 2013), we further verified the function of the key structural gene *ScSCPL2*, which encodes a SCPL acyltransferase, by virus-induced gene silencing (VIGS) in cineraria leaves combined with HPLC-MS/MS. The results will provide new insight into the molecular basis of the polyacylated anthocyanin biosynthesis mechanism in higher plants and are of great significance for blue flower molecular breeding of ornamental plants.

## Materials and Methods

### Plant Material and Sampling

**FIGURE 1:**
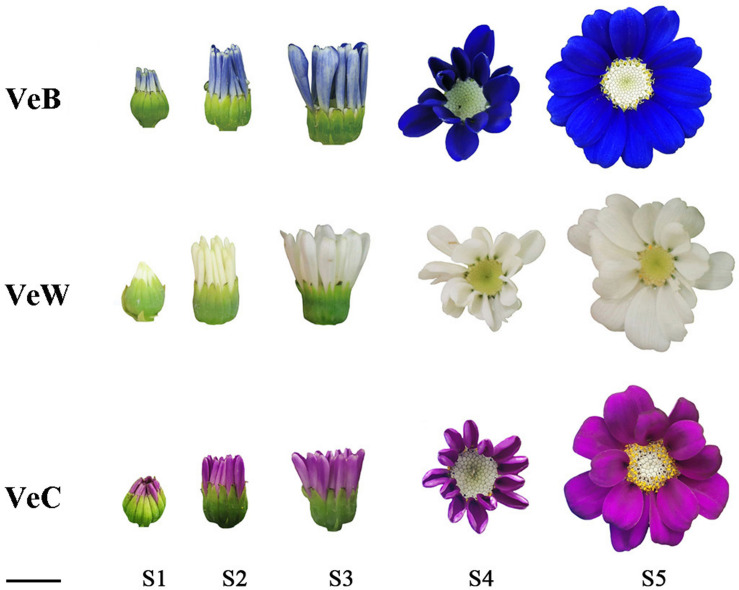
The five different developmental stages (S1–S5) of the VeB, VeW, and VeC capitulum. Bar, 1 cm.

The blue, white and carmine colours of cineraria cultivars ‘Venezia’ were chosen as plant materials, named VeB, VeW, and VeC, respectively, in this study. Seedlings were purchased from Youshang Flower Co., Ltd. (Shanghai). As the ray florets developed, five different stages were determined according to previous study (Jin et al., 2016). At the S1 stage, the ray florets outgrew the bract with colour, the length of the protruding bract was less than the length of the whole bract, and the capitulums were contracted. At the S2 stage, the length of the protruding bract was approximately 1.5 times the length of the bract, and the capitulums tightly overlapped. At the S3 stage, the ray florets were obviously elongated and widened, with the disc florets exposed for the first time. At the S4 stage, the outgrowing angle of the ray florets was approximately 45°, and the disc florets were completely visible. At the S5 stage, the ray florets were fully opened, and the outermost disc florets began spreading pollen (Figure 1). In this study, mixed samples of disc florets, ray florets, leaves and stems of VeB were used for full-length transcriptome sequencing. In addition, the ray florets at the S1, S2, S4, and S5 stages of VeB and VeW were used fornext-generation transcriptome sequencing, and three biological replicates were taken at each developmental stage. Then, the ray florets of VeB, VeC, and VeW at the S1, S2, S3, S4, and S5 stages were sampled for RT-PCR and qRT-PCR analysis.

### Identification of Anthocyanins by HPLC-MS/MS

Anthocyanin accumulation could accumulated in both leaves and ray florets of cineraria. In this study, the leaves and ray florets of VeB was used to identify anthocyanin components by HPLC-MS/MS analysis, and the ray florets of VeW and VeC were used as the control. Anthocyanin extraction and determination were performed as previously described (Sun et al., 2010). A total of 0.1 g of the sample was finely ground into powder in liquid nitrogen, and then homogenized in 5 ml of anthocyanin extracts [methanol: water: formic acid: trifluoroacetic acid (70:27:2:1, v/v/v/v)], leached at 4°C for 24 h in the dark. The supernatant volume was passed through a 0.22 μm filter after centrifugation. The sample was determined by the DIONEX high performance liquid chromatography equipped with a P680 HPLC pump, UltiMate 3000 auto-sampler, Thermostatted Column Compartment-100, and a TSK-GEL ODS-80Ts QA column (4.6 mm × 150 mm). The loading volume was 20 μL, mobile phase A was methanol:acetonitrile = 15:85 (v/v), mobile phase B was formic acid: water = 10:90 (v/v), column temperature was 25°C, flow rate was 1 mL/min. The gradient elution was performed as follows (A%/B%): 0–20 min, 70%–47%/30%–53%; 20–40 min, 47%/53%; 40–45 min, 47%–70%/53%–30%; 45–60 min, 70%/30%. Meanwhile, HPLC-ESI-MS/MS was used to analyze the anthocyanin structure of typical samples by Agilent 1100 LC/MSD Trap VL liquid chromatography-tandem mass spectrometry. The conditions of liquid chromatographic analysis were the same as above. The retention time and peak area of each sample was measured at wavelengths of 520 nm and in comparison with standards as cyanidin chloride (Sigma). Mass spectrometry analysis of each pigment components was mainly compared with the data of previous study (Sun et al., 2010).

### PacBio cDNA Library Construction, Sequencing and Data Analysis

A transcriptome provides a fast and economical method to systematically characterize gene models for one species without a genome reference. Recently, third-generation full-length transcriptome sequencing by PacBio SMRT has been widely used in transcriptome sequencing because it provides particularly long reads with high throughput and generates full-length transcripts (Li et al., 2020; Zhao et al., 2020). Generally, the protocol of transcriptome sequencing was including RNA extraction, transcriptome library construction and sequencing. Total RNA of the mixed samples was extracted using a Quick RNA Isolation Kit (Huayueyang Biotechnology Co. Ltd., Beijing, China). The purity and concentration of the RNA were assessed in a NanoDrop 2000 spectrophotometer. The third-generation full-length transcriptome library construction and sequencing were performed as previously described (Elmore et al., 2020). The main process of constructing a full-length transcriptome library was as follows: First-strand cDNA was synthesized from total RNA using a SMARTer polymerase chain reaction (PCR) cDNA Synthesis Kit (Clontech, United States), and then the first-strand cDNA was synthesized with SMARTScribe Reverse Transcriptase. Subsequently, a large amount of double stranded cDNA was produced. Full-length transcriptome library was sequenced by PacBio SMRT. According to the adapter in the sequence, all the original sequences were converted into circular-consensus sequence (CCS), and the sequences were divided into full-length sequences and non-full-length sequences according to whether there were 3′ primers, 5′ primers or PolyA in the CCS. Finally, consensus sequences were calibrated to get the high-quality sequences. All transcript sequences were annotated using the BLASTx alignment (*E*-value < 1 × 10^–5^) to the following database: Nr (National Center for Biotechnology Information non-redundant protein sequences), Pfam (Protein family), UniProtKB/Swiss-port (the UniProt Knowledgebase), COG (Clusters of Orthologous Groups), KOG (euKaryotic Ortholog Groups), GO (Gene Ontology); and KEGG (Kyoto Encyclopedia of Genes and Genomes pathway database). The best-aligning results from these databases were chosen to decide the sequence direction of the unigenes.

### Illumina cDNA Library Construction, Sequencing and Data Analysis

High throughput RNA sequencing (RNA-Seq) via next-generation sequencing technologies has been widely used in plants for comparative transcriptomics analysis (Lu et al., 2019; Pu et al., 2020). In this study, the total of RNA of the ray florets at the S1, S2, S4, and S5 stages of VeB and VeW was extracted using a Quick RNA Isolation Kit (Huayueyang Biotechnology Co. Ltd., Beijing, China). The main process of constructing the next-generation transcriptome library was as follows: magnetic beads with Oligo (dT) were used to enrich Eukaryote RNA, and the mRNA was mixed with the Fragmentation Buffer and fragmented randomly into short fragments. The first strand cDNA was synthesized with random hexamer primers. Subsequently, the second strand cDNA was synthesized using buffer, dNTPs, RNase H, and DNA polymerase I. The cDNA were purified with the AMPure XP system. The library was constructed through PCR amplification, and the quality was assessed on the Nanodrop and Agilent 2100 system. Finally, the next-generation transcriptome library was sequenced on Illumina HiSeq Platform. The generated final clean data were then mapped to the above high-quality transcript sequences obtained by third-generation full-length transcriptome sequencing. The expression level of each gene was calculated by using RSEM and converted into fragments per kilobase per million fragments (FPKM) according to the read counts (Li and Dewey, 2011).

### Differentially Expressed Transcript Analysis

To obtain differentially expressed transcripts of ray florets in VeB and VeW at the different developmental stages, the differentially expressed genes (DEGs) analysis between B1 and W1, B2 and W2, B4 and W4, B5 and W5 samples were carried out by DESeq package. These DEGs were identified by false discovery rate (False Discovery Rate, FDR) < 0.01 and a fold change ≥2 and the adjusted *P*-value < 0.05 were assigned (Anders and Huber, 2010). Differentially expressed genes were classified into functional categories using Blast2GO with the cut-off of *E*-value < 1 × 10^–5^ based on three levels of GO terms: biological processes (BP), molecular function (MF), and cell components (CC) (Conesa et al., 2005). DEGs were also assigned a candidate ko number through searching the KEGG pathway database using the online tool KAAS^1^ with the cut-off of *E*-value < 1 × 10^–5^ (Moriya et al., 2007). GO and KEGG enrichment analysis of DEGs was performed using the GOseqR packages^2^ and KOBAS^3^, respectively. Multiple Experiment Viewer software was used for analyzing the expression pattern of DEGs. The Euclidean distance was used to perform K-means cluster analysis on 1,066 differential genes, which differentially expressed in ray florets between VeB and VeW at the each developmental stages (S1, S2, S4, and S5), and the clusters positively correlated with the change of anthocyanin content were selected as key candidate clusters (Lin et al., 2018). The R language software package DCGL was used to analyze the genes in the key candidate clusters by WGCNA (Yang et al., 2013; Chen et al., 2016), and the genes related to anthocyanin metabolism were chosen to construct a co-expression network, and Cytoscape software^4^ was used to visualize this gene co-expression network (Doncheva et al., 2018).

### ScGT/AT Gene Screening and Phylogenetic Analysis

Genes encoding uridine diphosphate glycosyltransferase, GH1 family members and SCPL acyltransferase are key genes involved in polyacylation modification in blue cineraria. A total of 41 full-length sequences of *ScUGTs*, 25 full-length sequences of *ScGH1s* (including 11 full-length sequences of *ScAGGTs*), and 21 full-length sequences of *ScSCPLs* were obtained from the full-length transcriptome database in this study and translated using the NCBI Open Reading Frame Finder. Then, sequence alignments were performed using the MUSCLE algorithm in Molecular Evolutionary Genetics Analysis version X (MEGAX) with all *Arabidopsis thaliana UGTs*, *BGLUs*, *SCPLs*, and some amino acid sequences from other plants. Phylogenetic trees of *ScUGTs*, *ScGH1s*, and *ScSCPLs* were subsequently constructed according to the maximum likelihood (ML) estimation method. Tree nodes were evaluated using the bootstrap method for 1,000 replicates (Felsenstein, 1985). Evolutionary distances were computed using the *p*-distance method and expressed in units of amino acid differences per site. All positions containing gaps and missing data were eliminated prior to construction of the phylogenetic trees for the *ScUGT*, *ScGH1*, and *ScSCPL* genes. In addition, the expression patterns of some *ScUGTs*, *ScGH1s*, and *ScSCPLs*, which were clustered in the clades related to flavonoid modification (19 *ScUGTs*, 11 *ScAGGTs*, and 3 *ScSCPLs*), were analysed by RT-PCR.

### RT-PCR and qRT-PCR Analysis

In order to select the key candidate genes which only highly expressed in ray florets of blue cineraria cultivar VeB, the total RNA of ray florets of the five developmental stages in VeW, VeB, and VeC were extracted using Plant RNA Extraction Kit (HUAYUEYANG Biotechnology, Beijing, China). Then RNA was used to synthesize cDNA for RT-PCR and qRT-PCR with the transcription kit. The specific procedure for RT-PCR refered to the method of previous study (Li et al., 2019). Primers were designed by Primer 5 and the sequences were listed in Supplementary Table 1. The results of RT-PCR were detected by 1% agarose gel electrophoresis detection. Further expression analysis of highly expressed genes in the ray florets of VeB selected through RT-PCR were conducted by qRT-PCR. qRT-PCR analysis was performed on a CFX96 real-time system (Bio-Rad Laboratories, Hercules, CA, United States) according to the SYBR Premix Ex Taq kit (Japan, Takara) with three replicates. The results of qRT-PCR were read automatically by Bio-Rad CFX Manager and was performed using the 2^–Δ^ ^Δ^ ^*CT*^ method (Livak and Schmittgen, 2001). The primer information is listed in Supplementary Table 2.

### Transient Silencing of *ScSCPL2* Expression in Blue *S. cruentus* by VIGS

As the anthocyanin components of leaves were the same as those in ray florets of blue cineraria cultivar VeB, the VIGS system of cineraria leaves was used to quickly verify the function of the candidate structural gene *ScSCPL2*, which was performed according to the procedures described by previous study (Li et al., 2019). Gene sequences were identified from the full-length transcriptome sequencing database. A 368 bp fragment from the specific nucleotide region of *ScSCPL2* was amplified with specific primers (Supplementary Table 3). This PCR product was cloned into pTRV2 to form the pTRV2::*ScSCPL2* construct. While pTRV2::*ScANS* was used as positive control (Li et al., 2019). The pTRV2::*ScSCPL2* and pTRV2::*ScANS* constructs, as well as the pTRV1 which was essential for viral movement (Ziegler-Graff et al., 1991) and pTRV2 vectors, were transformed into *Agrobacterium tumefaciens* strain GV3101 by the freeze-thaw method. The transformed *A. tumefaciens* lines were grown in LB and resuspended to OD_600_ = 1.5 in infiltration buffer. A mixture of *A. tumefaciens* cultures containing pTRV1 and pTRV2 (or pTRV2::*ScSCPL2* or pTRV2::*ScANS*) was mixed in a 1:1 volume ratio before infection.

The infiltration mixture was introduced into 2-leaf stage seedlings by vacuum infiltration (VI). Ten days after the first VI infection, the second infection was carried out through leaf and shoot apical meristem syringe injection (SI), in which the infiltration mixture was introduced into the surfaces until the water-stained area accounted for more than 2/3 of the leaf. After each infection, the seedlings were cultured in the dark at 10°C for 2 d and 15°C for 1 d and then cultured in an illumination incubator at 20°C and 60% relative humidity for 12 h light/12 h dark. We observed the seedlings every 3 d for 30 d.

After the occurrence of the silenced phenotype, silenced tissues were sampled, and PCR was performed to detect the expression of the recombinant plasmid. The changes in anthocyanins in silenced tissues were analysed by HPLC-MS/MS to elucidate the function of *ScSCPL2* in polyacylated anthocyanin biosynthesis, and the specific operation procedure was the same as that described above.

## Results

### Characteristics of Anthocyanin Components in Leaves and Ray Florets of White, Carmine, and Blue *S. cruentus*

**FIGURE 2:**
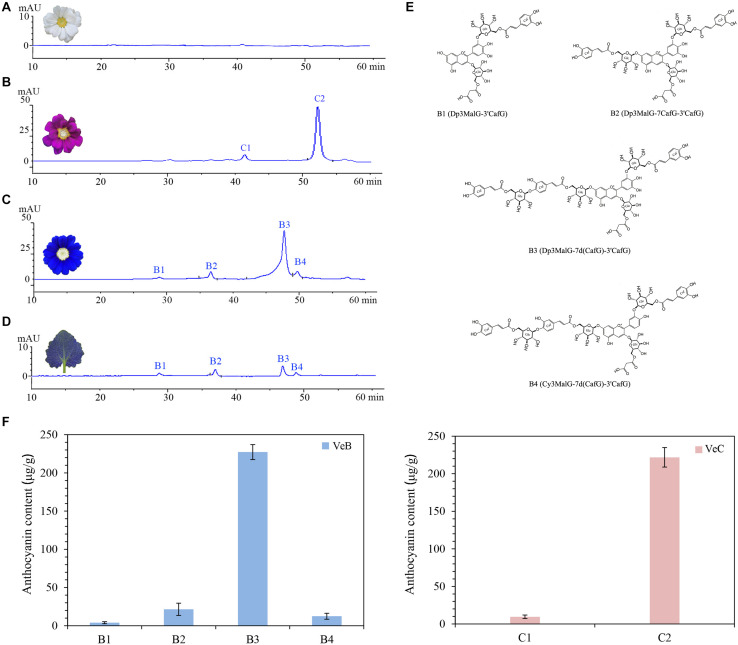
High-performance liquid chromatography (HPLC) analysis (520 nm) of anthocyanin components in the ray florets of VeW **(A)**, VeC **(B)**, and VeB **(C)**. **(D)** Anthocyanin composition analysis of the leaves of VeB. **(E)** Hypothetical chemical structures of B1, B2, B3, and B4 peaks. **(F)** The content of anthocyanins in the ray florets of VeB and VeC.

Cineraria is a rare plant species with pure and stable blue flowers in nature. In this study, HPLC-MS/MS was used to analyse the pigment composition of VeB, VeC, and VeW to explore the chemical basis of the blue colour of ray florets. We found that four different types of anthocyanins, B1, B2, B3, and B4, existed in the ray florets of VeB (Figure 2C). Two different types of anthocyanins, C1 and C2, existed in the ray florets of VeC (Figure 2B), while no anthocyanins were detected at 520 nm in VeW (Figure 2A).

MS data showed that the [delphinidin]^+^ fragment ion of m/z 303 was existed in B1, B2, and B3 peaks. Among them, B3 peak generated fragments at m/z 1,523 ([M + H]^+^), m/z 1,199 (loss of one glucosyl and one caffeoyl), m/z 875 (loss of two glucosyls and two caffeoyls), m/z 551 (loss of three glucosyls and three caffeoyls), which were consistent with the described for cinerarin (Goto et al., 1984; Sun et al., 2010). It was identified as Dp3MalG-7d(CafG)-3′CafG, which contained both 3′ and 7-polyacylated modifications (Figure 2E). B1 peak generated fragments at m/z 875 ([M + H]^+^), m/z 627 (loss of one glucosyl and one malonyl), m/z 551 (loss of one glucosyl and one caffeoyl). B2 peak generated fragments at m/z 1,199 ([M + H]^+^), m/z 875 (loss of one glucosyl and one caffeoyl), 551 (loss of two glucosyls and two caffeoyls) (Supplementary Table 4). Based on MS data, UV-vis absorption spectra and published literature (Sun et al., 2010), B1 and B2 peaks were tentatively postulated to be Dp3MalG-3′CafG and Dp3MalG-7CafG-3′CafG, respectively (Figure 2E). Besides, B4 peak contained a [cyanidin]^+^ fragment ion of m/z 287. It also generated fragments at m/z 1,507 ([M + H]^+^), m/z 1,183 (loss of one glucose and one caffeoyl), m/z 859 (loss of two glucoses and two caffeoyls), m/z 535 (loss of three glucoses and three caffeoyls), which were similar to the fragments of B3 peak. We speculated the chemical structure of B4 was Cy3MalG-7d(CafG)-3′CafG (Figure 2E). Quantitation of these four anthocyanins showed that the content of Dp3MalG-7d(CafG)-3′CafG was significantly higher than that of Dp3MalG-3′CafG and Dp3MalG-7CafG-3′CafG (Figure 2F). Thus, it was regarded as the main pigment component determining the blue colour of ray florets of cineraria. Furthermore, as C1 and C2 peaks contained the [cyanidin]^+^ fragment ion of m/z 287 based on MS data (Supplementary Table 4), they were regarded as cyanidin derivatives in this study. Quantitation of these two anthocyanins showed that C2 (221.84 μg/g) was the main pigment component determining the carmine colour of ray florets of cineraria (Figure 2F).

In addition, the leaves of VeB have the same colour as the ray florets. Exploring the consistency of the pigment components in the leaves and the ray florets will help us to perform functional verification of polyacylated genes in blue cineraria leaves by VIGS. HPLC-MS/MS was used to analyse the pigment components of the leaves of VeB, and four peaks also existed (Figure 2D). The UV-vis absorption maxima and protonated molecular data for these four peaks were consistent with those of anthocyanins detected in ray florets, which indicated that the anthocyanin components of leaves were the same as those in ray florets of VeB.

### Transcriptome Sequencing and Comparative Transcriptome Analysis

To identify the key genes for the biosynthesis of polyacylated anthocyanin in blue cineraria, RNA samples of mixed ray florets, leaves and stems were sequenced by PacBio SMRT. A total of 28.1 Gb clean data were obtained eventually. Based on the parameter of full passes ≥1 and sequence accuracy >0.9, 335, and 321 CCS were extracted from the original sequences, of which 277,604 were full-length non-chimeric sequences (FLNC), accounting for 82.79% of the total CCS (Supplementary Figure 1). A total of 32,199 high-quality consensus sequences were obtained by clustering the full-length non-chimeric sequences. The low-quality consensus sequences were corrected by the next-generation transcriptome data. Then, they were merged with the high-quality consensus sequence to perform de-redundancy analysis, and 19,469 transcript sequences were eventually obtained. These transcript sequences were compared with the databases NR, SwissProt, eggNOG, COG, CO, KOG, KEGG, and Pfam, and a total of 18,882 sequences were annotated (Supplementary Table 5).

**FIGURE 3:**
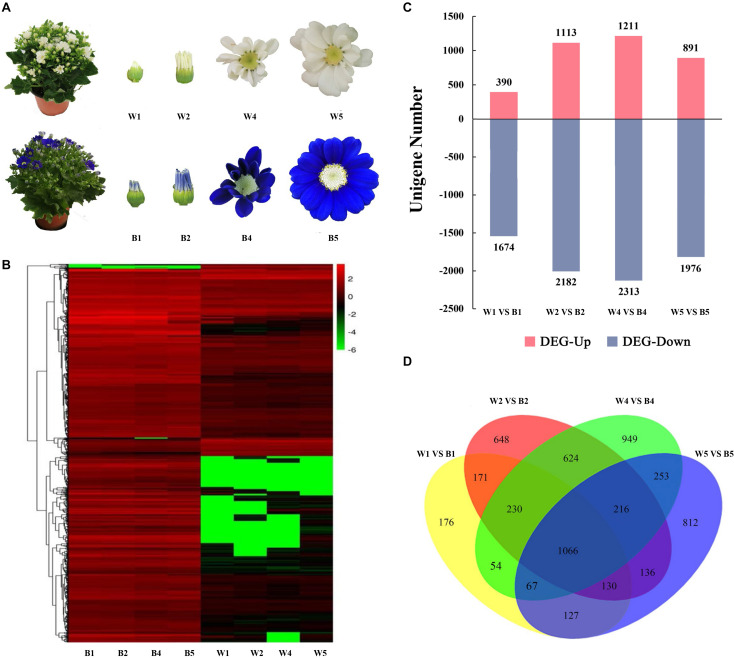
Comparative transcriptomics analysis between the ray florets of VeB and VeW. **(A)** The ray florets of VeW and VeB at the S1, S2, S4, and S5 stages (renamed W1, W2, W4, W5 and B1, B2, B4, B5, respectively) were used for second-generation sequencing. **(B)** Heatmap of all gene expression based on the second-generation sequencing data. **(C)** The number of up-regulated and down-regulated DEGs in comparisons among W1 vs B1, W2 vs B2, W4 vs B4, and W5 vs B5. **(D)** Venn diagram showing the number of DEGs through paired comparison.

In addition, the ray florets of VeW and VeB at the different developmental stages (S1, S2, S4, and S5) were analysed by next-generation sequencing (Figures 3A,B). The non-redundant transcripts measured by full-length transcriptome sequencing were used as a reference for sequence alignment and subsequent analysis. Sequence alignment between clean reads obtained by next-generation sequencing and transcripts through STAR revealed that the average alignment rate of clean reads that can be mapped to the non-redundant transcripts was 69.44%, while the average alignment rate of clean reads that contained the unique position in the transcripts was 47.14%, indicating that the transcriptome data were used efficiently in this study. Then, the transcriptome data of ray florets between VeW and VeB at the S1, S2, S4, and S5 stages were compared (W1 vs B1; W2 vs B2; W4 vs B4; and W5 vs B5), and 2,064, 3,295, 3,524, and 2,867 differentially expressed genes (DEGs) were identified and annotated, respectively (Figure 3C). These four groups of DEGs were intersected to screen 1,066 genes that were differentially expressed in VeW and VeB ray florets at the S1, S2, S4, and S5 stages simultaneously (Figure 3D).

**FIGURE 4:**
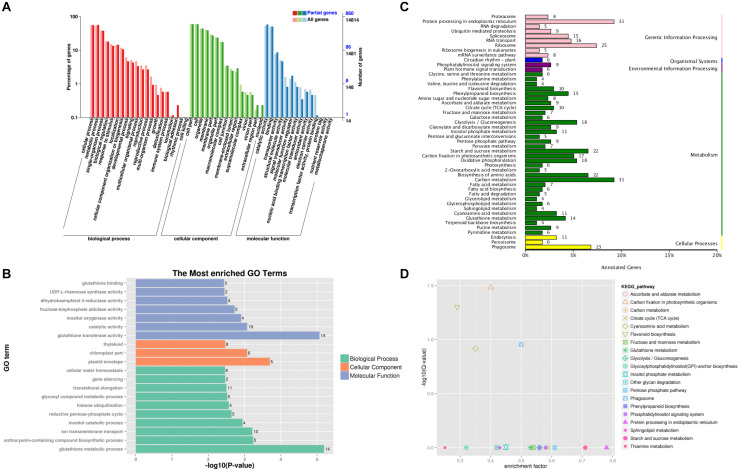
Gene Ontology (GO) and Kyoto Encyclopedia of Genes and Genomes (KEGG) pathway functional classification and enrichment analysis of the 1,066 differentially expressed genes (DEGs) identified from Venn diagram. **(A)** Functional classification of DEGs based on GO categorization. **(B)** GO enrichment analysis of the DEGs. **(C)** Pathway assignment based on the KEGG classification metabolism categories. **(D)** KEGG pathway enrichment analysis of the DEGs.

Gene Ontology (GO) enrichment analysis was used to define the function of the DEGs. A total of 1,066 DEGs were classified into 18, 15, and 11 categories and assigned to BP, cellular components, and MFs, respectively (Figure 4A). Proteins related to glutathione metabolic process from BP, plastid envelope from cellular component, and glutathione transferase activity from MF were the most enriched GO terms (Figure 4B). To further understand the biological functions of the 1,066 DEGs, KEGG enrichment analysis was used for pathway classification and functional enrichment. The pathways with the most DEG enrichment were carbon metabolism (ko01200), protein processing in endoplasmic reticulum (ko04141), and ribosome (ko03010). In addition, some DEGs were also enriched in flavonoid biosynthesis (ko00941) (Figures 4C,D).

**FIGURE 5:**
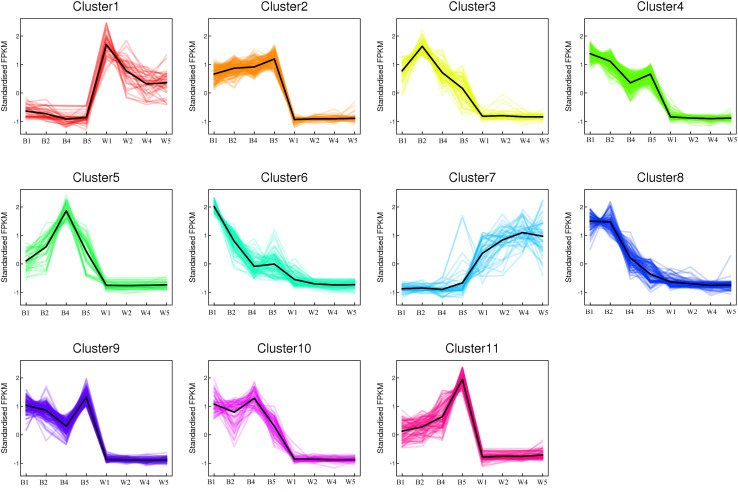
K-means analysis of 1,066 DEGs identified from Venn diagram. A total of 11 clusters were identified based on the expression profiles of these genes.

A total of 1,066 differentially expressed transcripts were divided into 11 clusters by K-means cluster analysis based on the expression profiles of these genes. The results revealed that the expression modes of genes in cluster 3, cluster 6, and cluster 8 appeared to be significantly positively correlated with the changes in anthocyanin content in VeW and VeB (Figure 5). A large number of structural genes and transcription factors associated with anthocyanin metabolism were found from these three clusters, including structural genes (*ScCHSs*, *ScF3Hs*, *ScF3*′*5*′*H*, *ScDFR*, *ScANS*, *ScMT*, and *ScGSTs*), transcription factors (*ScMYB*s and *ScbHLHs*) and modified genes (*ScUGTs*, *ScGH1s*, and *ScSCPLs*), which may be involved in the polyacylation modification of blue cineraria (Table 1).

**TABLE 1:** Stuctural genes and transcription factors associated with anthocyanin metabolism in 3, 6, and 8 clusters.

Cluster	#ID	B1 FPKM	B2 FPKM	B3 FPKM	B4_FPKM	W1 FPKM	W2_FPKM	W3_FPKM	W4_FPKM	nr_annotation	Abbreviation
cluster3	F01_transcript/20170	412.61	1,056.81	378.29	0.08	79.96	183.43	3.62	4.81	RecName: Full = Chalcone synthase; AltName: Full = Naringenin-chalcone synthase [*Callistephus chinensis*]	*CHS*
	F01_transcript/20876	49.10	61.44	45.76	49.70	19.53	20.26	18.90	22.18	Bifunctional dihydroflavonol 4-reductase/flavanone 4-reductase isoform 1 [*Theobroma cacao*]	*DFR*
	F01_transcript/17805	3,185.37	3,734.92	2,949.63	434.19	56.43	21.04	21.82	23.60	hypothetical protein [*Delphinium grandiflorum*]	*ScGH1-9*
	F01_transcript/17421	47.10	71.15	32.83	41.27	0.23	0.13	0.14	0.37	serine carboxypeptidase precursor [*Matricaria chamomilla*]	*ScSCPL3*
	F01_transcript/17893	93.60	128.50	66.00	44.17	0.04	0.07	0.05	0.25	PREDICTED: UDP-glucose 6-dehydrogenase 3 [*Nicotiana sylvestris*]	*ScUGT2*
	F01_transcript/14067	118.81	184.96	118.06	98.38	7.95	17.71	17.80	8.08	PREDICTED: probable methyltransferase PMT15 [*Nicotiana sylvestris*]	*MT*
cluster6	F01_transcript/15677	9.59	5.74	3.75	2.63	0.42	0.35	0.37	0.35	Putative methyltransferase family protein [*Solanum demissum*]	*MT*
	F01_transcript/14664	14.99	4.50	5.99	1.23	0.00	0.00	0.00	0.00	PREDICTED: probable methyltransferase PMT19 [*Nelumbo nucifera*]	*MT*
	F01_transcript/14764	101.69	37.18	7.36	1.01	2.31	0.09	0.00	0.05	PREDICTED: beta-glucosidase 11-like isoform X1 [*Vitis vinifera*]	*ScGH1-2*
	F01_transcript/14853	195.30	78.52	27.49	1.25	3.35	0.26	0.00	0.00	unnamed protein product [*Coffea canephora*]	*ScGH1-3*
	F01_transcript/16506	12.23	8.72	2.62	2.80	2.53	0.74	0.25	0.44	PREDICTED: glucan endo-1,3-beta-glucosidase 1 [*Nicotiana sylvestris*]	*ScGH1-4*
	F01_transcript/18027	335.20	281.57	102.18	5.34	1.98	0.48	0.00	0.35	hypothetical protein [*Delphinium grandiflorum*]	*ScGH1-10*
	F01_transcript/29185	70.47	42.36	21.38	16.51	1.21	0.28	0.08	0.07	PREDICTED: ultraviolet-B receptor UVR8 [*Populus euphratica*]	*UVR8*
	F01_transcript/26228	177.84	137.87	52.74	3.26	189.47	83.97	22.78	40.49	PREDICTED: myb-related protein 308-like [*Populus euphratica*]	*ScMYB1*
	F01_transcript/25625	826.38	618.56	283.88	36.04	754.49	212.47	24.92	148.17	transcription factor GbMYB1 [*Gynura bicolor*]	*ScMYB3*
cluster8	F01_transcript/8489	100.91	72.29	26.33	2.14	2.48	0.82	0.83	0.34	dihydroflavonol 4-reductase [*Pericallis cruenta*]	*DFR*
	F01_transcript/22631	602.84	405.08	218.02	3.77	19.40	0.53	0.10	0.02	anthocyanidin synthase [*Pericallis cruenta*]	*ANS*
	F01_transcript/3423	63.99	52.63	18.67	0.29	17.90	13.28	0.76	4.21	flavanone 3-hydroxylase [*Gynura bicolor*]	*F3H*
	F01_transcript/21258	1,353.22	1,239.51	919.56	39.43	344.68	232.82	38.25	114.85	flavanone 3-hydroxylase [*Gynura bicolor*]	*F3H*
	F01_transcript/10531	1,004.11	908.97	348.96	11.52	71.63	7.37	0.33	1.58	flavanone 3-hydroxylase [*Gynura bicolor*]	*F3H*
	F01_transcript/21783	1,389.48	1,093.78	453.99	55.81	45.46	10.89	17.24	5.74	dihydroflavonol reductase [*Gynura bicolor*]	*DFR*
	F01_transcript/21611	1,500.68	1,171.75	668.33	64.66	1.86	1.16	3.78	0.26	dihydroflavonol reductase [*Gynura bicolor*]	*DFR*
	F01_transcript/23062	1,478.20	1,228.58	726.19	51.23	61.72	15.91	28.77	7.96	dihydroflavonol reductase [*Gynura bicolor*]	*DFR*
	F01_transcript/18084	3,611.16	3,329.84	2,576.61	119.82	85.51	4.41	1.83	0.94	PREDICTED: serine carboxypeptidase-like[*Nicotiana tomentosiformis*]	*ScSCPL2*
	F01_transcript/17059	4,165.92	2,916.19	1,632.97	90.84	104.27	3.44	1.34	1.56	glycoside hydrolase family 1 [*Artemisia annua*]	*ScGH1-5*
	F01_transcript/17114	295.91	105.11	33.61	3.82	9.74	0.36	0.04	0.00	glycoside hydrolase family 1 [*Artemisia annua*]	*ScGH1-6*
	F01_transcript/17805	55.97	67.27	54.70	7.74	0.06	0.00	0.00	0.31	beta-glucosidase 11 isoform X4 [*Helianthus annuus*]	*ScGH1-11*
	F01_transcript/18352	380.81	536.20	749.78	111.22	0.18	0.00	0.46	0.00	beta-glucosidase 11-like isoform X3 [*Lactuca sativa*]	*ScGH1-12*
	F01_transcript/7187	74.91	53.76	20.34	2.03	0.38	0.07	0.02	0.14	PREDICTED: beta-glucosidase 22-like [*Elaeis guineensis*]	*ScGH1-13*
	F01_transcript/17811	847.47	837.09	337.54	6.91	20.62	0.85	0.21	0.95	PREDICTED: crocetin glucosyltransferase, chloroplastic-like [*Vitis vinifera*]	*ScUGT1*
	F01_transcript/17925	688.85	704.08	386.07	12.27	18.22	0.45	0.28	0.47	PREDICTED: crocetin glucosyltransferase, chloroplastic-like [*Vitis vinifera*]	*ScUGT3*
	F01_transcript/20913	71.08	69.76	35.73	31.14	1.94	1.88	3.81	1.46	PREDICTED: probable beta-1,3-galactosyltransferase 10-like [*Glycine max*]	*ScUGT5*
	F01_transcript/18974	7.26	8.09	19.95	72.64	0.28	0.15	0.12	0.41	UDP-glycosyltransferase 73A17 [*Camellia sinensis*]	*ScUGT4*
	F01_transcript/16894	354.30	231.58	156.77	7.14	35.22	1.41	0.15	0.20	bHLH transcription factor [*Dahlia pinnata*]	*bHLH*
	F01_transcript/13375	202.60	166.73	66.03	10.36	5.47	1.44	1.00	2.12	bHLH transcription factor [*Dahlia pinnata*]	*bHLH*
	F01_transcript/23148	21.02	31.99	4.09	4.59	0.24	0.07	0.11	0.12	PREDICTED: transcription factor bHLH79 [*Prunus mume*]	*bHLH*
	F01_transcript/2735	60.56	54.51	19.62	1.72	5.54	0.25	0.03	0.04	bHLH transcription factor [*Dahlia pinnata*]	*bHLH*
	F01_transcript/6748	38.62	35.73	13.08	1.71	0.57	0.29	0.20	0.35	bHLH transcription factor [*Dahlia pinnata*]	*bHLH*
	F01_transcript/1904	16.61	16.91	5.32	0.42	1.54	0.13	0.00	0.00	bHLH transcription factor [*Dahlia pinnata*]	*bHLH*
	F01_transcript/27268	12.13	40.92	16.35	0.25	1.60	0.10	0.62	1.01	Transcription repressor MYB4 -like protein [*Gossypium arboreum*]	*ScMYB2*
	F01_transcript/30445	980.63	349.26	71.79	0.29	1.43	0.14	0.14	0.18	PREDICTED: MYB-like transcription factor ETC1 [*Eucalyptus grandis*]	*ScMYB4*
	F01_transcript/18873	779.80	503.07	156.28	0.03	23.29	0.05	0.02	0.08	flavonoid-3’,5’-hydroxylase [*Pericallis cruenta*]	*F3’5’H*
	F01_transcript/24911	151.57	95.33	58.26	2.78	0.68	0.04	0.00	0.00	glutathione *S*-transferase [*Dahlia pinnata*]	*GST*
	F01_transcript/25545	3,672.06	4,691.52	2,440.66	195.28	182.62	63.23	22.85	13.72	PREDICTED: glutathione *S*-transferase-like [*Eucalyptus grandis*]	*GST*
	F01_transcript/26648	1,187.26	1,125.50	1,074.77	62.14	72.10	2.37	0.53	0.08	glutathione *S*-transferase [*Dahlia pinnata*]	*GST*
	F01_transcript/26883	1,013.47	628.40	452.50	21.19	31.43	1.48	0.19	0.42	glutathione *S*-transferase [*Dahlia pinnata*]	*GST*

### Identification of *ScGTs/ATs* Involved in Polyacylated Anthocyanin Biosynthesis

The alternation of glucosylation and caffeoylation catalysed by glucosyltransferase UGTs/*GH1*s and caffeoyl transferase SCPLs, respectively, at the 3′ or 7-position of anthocyanins ultimately leads to the formation of polyacylated cinerarin, which is crucial to blue ray floret coloration. To identify all genes encoding glycolyltransferase and acyltransferase that may be involved in anthocyanin polyacylation modification, we searched these three groups of structural genes from the third-generation transcriptome database and obtained a total of 41 full-length sequences of *UGTs*, 25 full-length sequences of *GH1s* (including 11 full-length sequences of *AGGTs*, which belong to *GH1*, catalyse the glycosylation of anthocyanins with acyl-glucoses as glucosyl donors), and 21 full-length sequences of *SCPLs*.

**FIGURE 6:**
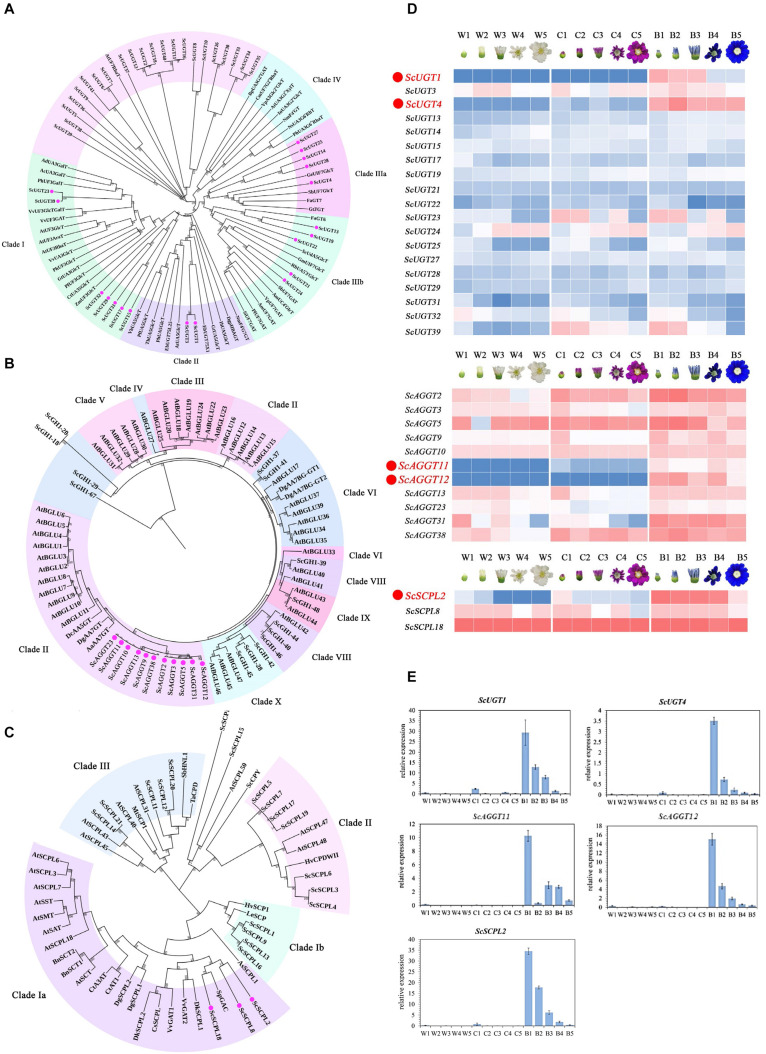
Identification of *ScUGTs/ScGH1s/ScSCPLs* involved in polyacylated anthocyanin biosynthesis by phylogenetic analysis and gene expression pattern analysis. Amino acid sequences of *ScUGTs* **(A)**, *ScGH1s* **(B)**, *ScSCPLs* **(C)**, and all arabidopsis UGTs/BGLUs/SCPLs and some sequences from other plants were used to build three phylogenetic trees based on maximum likelihood. **(D)** Heatmap analysis based on the RT-PCR data was performed to screened out the candidate key genes (*ScUGTs/ScAGGTs/ScSCPLs*) encoding glycolyltransferase and acyltransferase that might be involved in the polyacylation modification of anthocyanins. **(E)** The expression profiles of *ScUGT1*, *ScUGT4*, *ScAGGT11*, *ScAGGT12*, and *ScSCPL2* were analyzed by qRT-PCR. The data were the mean ± SD from three biological replicates.

Phylogenetic analysis of 41 *ScUGTs* revealed that 19 of them were clustered in four clades related to flavonoid glycosylation. *ScUGT15*, *ScUGT17*, *ScUGT23*, *ScUGT29*, *ScUGT31*, *ScUGT32*, and *ScUGT39* were clustered in Clade I with the genes encoding anthocyanin 3-*O*-glucosyltransferase in *Arabidopsis thaliana*, *Vitis vinifera*, and *Gentiana triflora* (Figure 6A), which may be involved in modifying the glycosylation of the 3-*O*-position of anthocyanins in cineraria. *ScUGT1* and *ScUGT3* were clustered with the genes identified from *Perilla frutescens*, *Verbena hybrida*, *P. hybrid*, and *G. triflora* in Clade II (Figure 6A), which were related to the glycosylation of the 5-*O*-position of anthocyanins. These genes were also clustered with *DgpHBAGT*, which is involved in the synthesis of *p*-hydroxybenzoyl-glucose (pHBG), which acts as a zwitter donor in acylation and glucosylation in *D. grandiflorum* (Nishizaki et al., 2014). *ScUGT4*, *ScUGT14*, *ScUGT25*, *ScUGT27*, and *ScUGT28* were clustered with *Gt3*′*GT* and *SbUF7GlcT* in Clade IIIa (Figure 6A), which might be involved in the glycosylation of the 3′-*O*-position of cinerarin. In addition, *ScUGT13*, *ScUGT19*, *ScUGT21*, *ScUGT22*, and *ScUGT24* were clustered with *VpUF7GAT*, *PfUF7GAT*, and *AmUF7GAT* in Clade IIIb (Figure 6A), which specifically catalysed the glycosylation of the 7-*O*-position of flavones with UDP-glucuronic acid as the glycosyl donor (Ono et al., 2010). Phylogenetic analysis of 25 *ScGH1s* showed that *ScAGGT2*, *ScAGGT3*, *ScAGGT5*, *ScAGGT9*, *ScAGGT10*, *ScAGGT11*, *ScAGGT12*, *ScAGGT13*, *ScAGGT23*, *ScAGGT31*, and *ScAGGT38* were clustered with AtBGLU10, DgAA7GT, DcAA5GT, and AaAA7GT (Figure 6B), which catalysed the glycosylation of the 5- or 7-*O*-position of anthocyanins in Clade I, which indicated that these *ScAGGTs* might be involved in the glycosylation of the 7-*O*-position of cinerarin.

In addition, phylogenetic analysis of 21 *ScSCPLs* showed that *ScSCPL2*, *ScSCPL8*, and *ScSCPL18* were clustered in Clade Ia with genes encoding flavonoid acyltransferase in *C. ternatea*, *A. thaliana*, and *D. grandiflorum* (Figure 6C) (Noda et al., 2006; Fraser et al., 2007; Nishizaki et al., 2013; Shi and Xie, 2014). *ScSCPL1*, *ScSCPL9*, *ScSCPL13*, and *ScSCPL16* were clustered in Clade Ib (Figure 6C), and these genes might be related to the plant wound response (Moura et al., 2001). *ScSCPL3*, *ScSCPL4*, *ScSCPL5*, *ScSCPL6*, *ScSCPL7*, *ScSCPL17*, and *ScSCPL19* were clustered in Clade II (Figure 6C), of which the members play an important role in plant growth and development, such as brassinolide signalling and seed germination (Li et al., 2001). *ScSCPL11*, *ScSCPL12*, *ScSCPL14*, and *ScSCPL21* were clustered in Clade III (Figure 6C), however, the function of this clade is not clear.

The expression patterns of some *ScUGTs*, *ScAGGTs*, and *ScSCPLs*, which were clustered in the clades related to flavonoid modification (19 *ScUGTs*, 11 *ScAGGTs*, and 3 *ScSCPLs*), were analysed by RT-PCR. The results showed that *ScUGT23* and *ScUGT39*, belonging to Clade I *UGTs*, were highly expressed in the ray florets of VeB and at a high expression level in VeC but almost not expressed in VeW (Figure 6D), which indicated that *ScUGT23* and *ScUGT39* might be involved in the primary glycosylation of 3-*O*-position of anthocyanin in cineraria. *ScUGT1* clustered with *DgpHBAGT* in Clade II, was highly expressed in VeB and might be involved in the synthesis of caffeoylglucose. *ScUGT4* clustered with *Gt3*′*GT* in Clade IIIa was highly expressed in VeB but almost not expressed in VeW and VeC (Figure 6D), which indicated that it might be involved in the glycosylation of 3′-*O*-position of cinerarin. RT-PCR analysis also revealed that *ScAGGT11* and *ScAGGT12* clustered with *DgAA7GT* and *AaAA7GT* in Clade I highly expressed in VeB but slightly expressed in VeC and VeW (Figure 6D), which indicated that *ScAGGT11* and *ScAGGT12* might be involved in the glycosylation of the 7-*O*-position of cinerarin.

**FIGURE 7:**
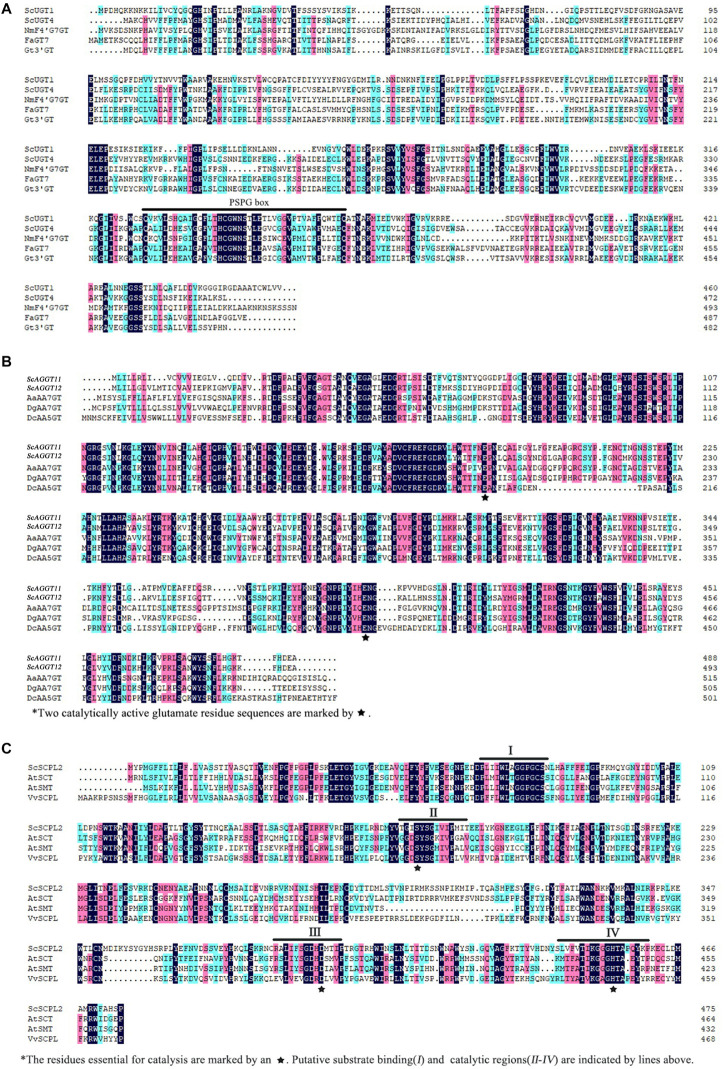
Multiple alignments of amino acid sequences of *ScUGT1*, *ScUGT4*, *ScBGLU11*, *ScBGLU12*, and *ScSCPL2*. **(A)** Sequence alignment of *ScUGT1* and *ScUGT4* (*Senecio cruentus*), *NmF4*′*G7GT* (*Nemophila menziesii*), *FaGT7* (*Fragaria ananassa*), and *Gt3*′*GT* (*Gentiana triflora*). **(B)** Sequence alignment of *ScAGGT11* and *ScAGGT12* (*Senecio cruentus*), *AaAA7GT* (*Agapanthus africanus*), *DgAA7GT* (*Delphinium grandiflorum*), and *DcAA5GT* (*Dianthus caryophyllus*). **(C)** Sequence alignment of *ScSCPL2* (*Senecio cruentus*), *AtSCT (Arabidopsis thaliana)*, *AtSMT* (*Arabidopsis thaliana*), and *VvSCPL* (*Vitis vinifera*).

In terms of acylation modification of cineraria, *ScSCPL2*, which belonged to Clade Ia, was highly expressed in the ray florets of VeB, slightly expressed in VeC at the S1 stage, and almost not expressed in VeC at the other developmental stages or in VeW. Furthermore, the expression levels of *ScSCPL8* and *ScSCPL18*, which also belonged to Clade Ia, were not significantly different among VeB, VeC, and VeW (Figure 6D). These results indicated that *ScSCPL2* might be involved in the acylation of blue cineraria. qRT-PCR verified the above results (Figure 6E). In addition, we found that these candidate modified genes (*ScUGT1*, *ScUGT4*, *ScAAGT11*, *ScAAGT12*, and *ScSCPL2*) all existed in cluster 8 of DEGs. Sequence analysis of ScUGT1 and ScUGT4 showed that their amino acid sequences shared the common domain of a PSPG box (putative secondary product glycosyltransferase) in the *C*-terminal region (Figure 7A). Meanwhile, two catalytically active glutamate residue sequences of ScAAGT11 and ScAAGT12 (Figure 7B) as well as putative substrate binding and catalytic regions of *ScSCPL2* were also identified and marked (Figure 7C).

### Transient Silencing of *ScSCPL2* Expression by VIGS in Blue Cineraria

**FIGURE 8:**
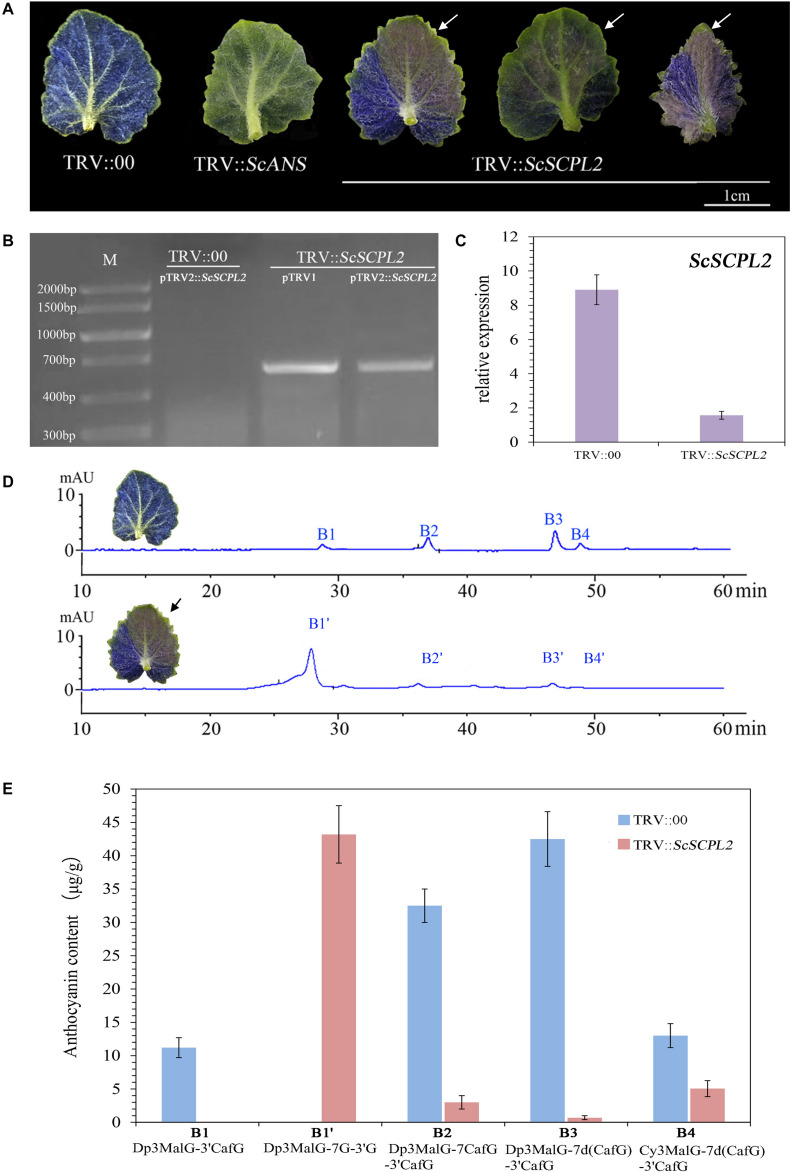
Transient silencing of *ScSCPL2* by VIGS in blue cineraria. **(A)** The phenotype of the leaves of blue cineraria whose *ScSCPL2* gene was silenced by VIGS. **(B)** Detection of pTRV1 and pTRV2::*ScSCPL2* in the silencing tissue of blue cineraria. **(C)** qRT-PCR analysis of *ScSCPL2* both in the silencing tissue and negative control. **(D)** Anthocyanin composition in the leaves and *ScSCPL2*-silenced tissues of blue cineraria by HPLC analysis. **(E)** The content of anthocyanins in the leaves and *ScSCPL2*-silenced tissues of blue cineraria.

As the anthocyanin components of leaves were the same as those in ray florets of VeB, the VIGS system of cineraria leaves was used to quickly verify the function of the candidate structural gene *ScSCPL2*. The vectors, including pTRV1, pTRV2, pTRV2::*ScANS* and pTRV2::*ScSCPL2*, were transformed into *A. tumefaciens* strain GV3101 by the freeze-thaw method. Then, the infiltration mixture containing pTRV1 and pTRV2 (or pTRV2::*ScANS* or pTRV2::*ScSCPL2*) was introduced into 2-leaf stage seedlings by VI. Among them, pTRV2::*ScANS* was used as a positive control to show the feasibility of this VIGS system (Li et al., 2019). The leaves of blue cineraria infiltrated with pTRV2::*ScSCPL2* exhibited a function-loss phenotype, whose colour turned pink, at 30 days post-infiltration. No leaves infiltrated with pTRV2 (regarded as a negative control) eventually exhibited a function-loss phenotype (Figure 8A). In addition, RT-PCR showed that the presence of pTRV2::*ScSCPL2* was only detected in *ScSCPL2*-silenced tissues (Figure 8B). Moreover, qRT-PCR results showed that the expression of *ScSCPL2* in the tissues with functional loss was significantly lower than that in the negative control (Figure 8C). These results confirmed that TRV vectors could infiltrate cineraria successfully and that pTRV2::*ScSCPL2* could knock down the expression of the endogenous *ScSCPL2* gene to make leaves appear pink.

Then we used HPLC-MS/MS to identify the anthocyanin composition and content of *ScSCPL2*-silenced tissues. Four peaks existed in these tissues, named B1′, B2′, B3′, and B4′ (Figure 8D). Among them, the retention time, UV-vis absorption maxima and protonated molecule results of B2′, B3′, and B4′ were the same as those of B2, B3, B4 in VeB (Supplementary Table 6). The absorption peak around 330 nm of B1’ was absent while it was existed in B1 (Supplementary Figure 2), which indicated absence of caffeoyl moiety in the molecule of B1′. In addition, we found that the B1′ peak contained a [delphinidin]^+^ fragment ion of m/z 303, and its molecular ion peak [M + H]^+^ was m/z 875, which was consistent with B1 (Dp3MalG-3′CafG, one glucosyl and one caffeoyl existed at the 3′ position of the B ring). Two intermediate fragment ions, m/z 551 ([Dp3MalG + H]^+^) and m/z 713 ([Dp3MalG-3′G + H or Dp3MalG-7G + H]^+^), were detected in B1′, while only one fragment ion of m/z 551 ([Dp3MalG + H]^+^) existed in B1, which indicated that a single glucosyl existed at the 3′ and 7-positions of B1′ simultaneously. Based on these UV-vis absorption spectra and protonated molecule data, we speculated the chemical structure of B1′ was Dp3MalG-7G-3′G. After inhibiting the expression of *ScSCPL2* in blue cineraria, the content of Dp3MalG-7CafG-3′CafG and Dp3MalG-7d(CafG)-3′CafG (cinerarin) decreased from 32.5to 3.01 μg/g and from 42.5 to 0.69 μg/g, respectively, while Dp3MalG-7G-3′G, which had no acylation at the 3′ or 7-position, accumulated at a high level (43.2 μg/g) (Figure 8E). These results showed that *ScSCPL2* was a key structural gene mainly involved in the 3′ and 7-position acylation for blue flower formation in cineraria.

### Identification of *MYBs* Involved in the Regulation of Polyacylated Anthocyanin Biosynthesis

**FIGURE 9:**
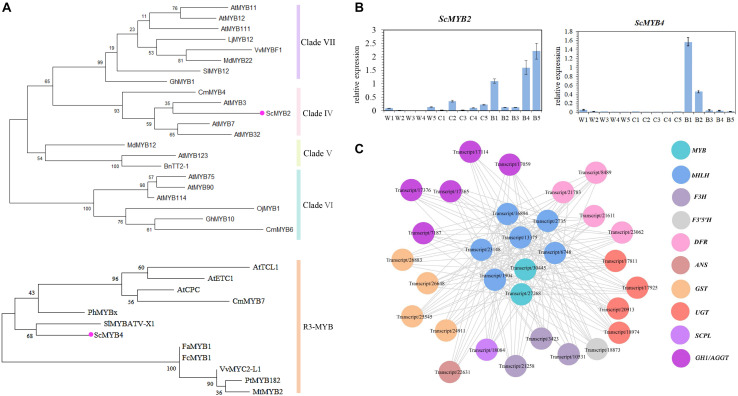
Identification of *MYBs* involved in the regulation of polyacylated anthocyanin biosynthesis using bioinformatics and qRT-PCR analysis. **(A)** Amino acid sequences of *ScMYB2* and *ScMYB4* and arabidopsis MYBs belonging to the Clade IV-VII and R3-MYB and some *MYB* sequences from other plants were used to build a phylogenetic tree based on maximum likelihood, which was employed to infer the MYB candidates of cineraria involved in polyacylation modification. **(B)** The expression profiles of *ScMYB2* and *ScMYB4* during the ray florets developed were confirmed by qRT-PCR. The data were the mean ± SD from three biological replicates. **(C)** The relationship between *ScMYB2*, *ScMYB4* and the key structural genes related to delphinidin biosynthesis and polyacylation modification of cineraria in cluster 8 of DEGs was analyzed by gene co-expression network. The circles represented the transcripts, and the grey lines represented connectivity among the transcripts.

The MYB-bHLH-WD40 complex plays an important role in regulating the transcription of structural genes involved in the anthocyanin biosynthesis pathway. The MYB transcription factor is extremely important in the MYB-bHLH-WD40 complex (Jaakola, 2013). According to the K-means analysis of differentially expressed genes, Two MYB transcription factors named *ScMYB2* and *ScMYB4* from cluster 8 were identified. Phylogenetic analysis indicated that *ScMYB2* belonged to subfamily 4 of the R2R3-MYB transcription factor gene family, while *ScMYB4* was clustered to the R3 MYB transcription factor gene family (Figure 9A). The expression profiles of these two MYB transcription factors were detected using qRT-PCR. The results showed that *ScMYB2* and *ScMYB4* were only highly expressed in VeB, while they were barely detectable in VeW and VeC (Figure 9B). In addition, *ScMYB2* and *ScMYB4* were found to have expression patterns closely related to key structural genes, such as *ScUGT1*, *ScUGT4*, *ScBGLU11*, *ScBGLU12*, and *ScSCPL2*, which might be involved in the polyacylation modification of cinerarin, through gene coexpression network analysis, and these genes all belonged to cluster 8 of DEGs (Figure 9C). Based on the above results, *ScMYB2* and *ScMYB4* were selected as key candidate transcription factors in the regulation of the delphinidin biosynthesis pathway and polyacylation modification in blue cineraria.

## Discussion

### *ScUGT1/4* and *ScAGGT11/ScAGGT12* Might Be Involved in the Glycosylation of the 3′- and 7-*O*-Position of Cinerarin

Glycosyltransferases and acyltransferases involved in anthocyanin modification resulted in rich variation in flower colour. Glycosylation and acylation at the 3′ position of the B ring and 7-position of the A and C rings play very important roles in the formation of stable blue flower colour (Matsuba et al., 2010; Nishizaki et al., 2013). Cineraria is an important potted ornamental plant, and its blue flowers contain 3′,7-polyacylated delphinidin-type anthocyanins, which has not been reported in any other plants. The main anthocyanin in the blue colour of the ray florets was cinerarin, which contained one glucosyl and one caffeoyl at the 3′ position, two glucosyls and two caffeoyls at the 7-position (Goto et al., 1984; Suzuki et al., 2003), suggesting the important role of polyacylation in blue flower formation.

Glycosylation by GTs provides the basis of acylation by ATs. UGTs catalyse flavonoids to biosynthesize various glycoside derivatives, among which 3-/5-position modifications are the common skeleton of anthocyanins, while UGTs that are specific for 3′ or 7-position modifications can catalyse polyacylated anthocyanin biosynthesis (Sasaki and Nakayama, 2015). In this study, using comparative transcriptome analysis combined with gene expression, we found that *ScUGT1* and *ScUGT4* were highly expressed in the ray florets of VeB but almost not expressed in the ray florets of VeW and VeC (Figures 6D,E). Phylogenetic analysis has revealed that the positional specificities of the glycosylation modification catalysed by UGTs are closely related to the phylogenetic clades of the enzymes. We found that *ScUGT1* was clustered with *DgpHBAGT*, which is involved in the synthesis of *p*-hydroxybenzoyl-glucose (pHBG), which acts as a zwitter donor in acylation and glucosylation in *D. grandiflorum* (Figure 6A) (Nishizaki et al., 2014). This result indicated that *ScUGT1* might be involved in the synthesis of caffeoylglucose, a type of acyl group donor, in cineraria. Meanwhile, both *ScUGT4* and *GtUA3*′*GT* belonged to Clade IIIa by phylogenetic analysis (Figure 6A). *GtUA3*′*GT* of *G. triflora* is involved in gentiodelphin (3G-5CafG-3′CafG) biosynthesis, and the *GtUA3*′*GT* knockout lines accumulate delphinidin 3G-5CafG as the major flower pigment instead of gentiodelphin (Tasaki et al., 2019). Thus, we selected *ScUGT4* as the key candidate gene that may catalyse the 3′ position glycosylation of cinerarin. However, the enzymatic function of ScUGT4 needs further verification.

Uridine diphosphate-dependent glycosyltransferase family 1 that specifically catalyse the 7-position glycosylation of anthocyanins have not yet been discovered, while UGTs that catalyse the 7-position glycosylation of other flavonoids have been characterized in blue *Nemophila menziesii* and *Veronica persica*. The 7-glycosylated flavonoids catalysed by *NmF4*′*G7GT* were used as anthocyanin copigments to produce a deeper and bluer colour through intramolecular stacking. The 7-glycosylated flavonoids catalysed by *VpUF7GAT* complexed with metal ions and glycosylated anthocyanins also make plants appear blue (Ono et al., 2010; Okitsu et al., 2018). AGGT is another glycosyltransferase involved in the modification of anthocyanins. For example, the 7-polyacylated anthocyanin of delphinidin was synthesized via step-by-step enzymatic reactions of glycosylation catalysed by BGLUs (*DgAA7GT* and *DgAA7BG-GT*) and *p*-hydroxybenzoylation catalysed by SCPL-AT (*DgSCPL2*) (Matsuba et al., 2010; Luang et al., 2013; Nishizaki et al., 2013). In this study, *ScAGGT11* and *ScAGGT12* were specifically highly expressed in the ray florets of VeB (Figures 6D,E). They are both clustered in the same clade as *DgAA7GT* (Figure 7B), which were speculated to be involved in the glycosylation of the 7-*O*-position of cinerarin in blue cineraria. However, the specific function needs further verification.

### *ScSCPL2* Participated in the 3′ and 7-Position Acylation of Cinerarin

There are two important gene families related to the acylation of anthocyanins: acyl-CoA-dependent acyltransferase BAHD and SCPL utilizing acyl-Glc as the donor molecule. The products of the BAHD family are mainly monoacylated anthocyanins (Bontpart et al., 2015). Suzuki et al. (2003) identified a BAHD-type AAT acyltransferase *Sc3MaT* in blue cineraria. The analysis of enzymatic properties showed that it specifically used malonyl-CoA as the donor and anthocyanin 3-*O*-glucoside as the acceptor to synthesize anthocyanin 3-*O*-glucose-6′′-malonyl (Anthocyanin-3GMal). However, the 3′ and 7-position aromatic acyl modifications of anthocyanins are key for the formation of bright blue flowers of cineraria. At present, there are no studies on the mechanism of 3′ and 7-position acylation of cineraria.

In this study, we found that *ScSCPL2*, encoding an acyl-Glc-dependent acyltransferase in cineraria, showed specific high expression in VeB through comparative transcriptomic analysis combined with gene expression (Figures 6D,E). VIGS showed that the leaves of cineraria infiltrated with pTRV2::*ScSCPL2* exhibited a function-loss phenotype, whose colour turned pink, at 30 days post-infiltration (Figure 8A). In addition, the content of Dp3MalG-7CafG-3′CafG and Dp3MalG-7d(CafG)-3′CafG (cinerarin) decreased significantly after inhibiting the expression of *ScSCPL2* in blue cineraria, while Dp3MalG-7G-3′G, which had no acylation at the 3′ or 7-position, accumulated at a high level (Figures 8D,E). Phylogenetic analysis revealed that *ScSCPL2* belonged to the Clade Ia subfamily (Figure 6C), whose members mainly encoded acyltransferases to catalyse the acylation of flavonoids (including anthocyanins). For example, *At2g23000*, a member of the Clade Ia subfamily in *A. thaliana*, has been shown to use 1-*O*-sinapoyl-β-D-glucoses as substrates to transfer sinapoyl groups to cyanins to form sinapoylated cyanins (Fraser et al., 2007; Shi and Xie, 2014). *CtA3*′*AT* of *C. ternatea* could participate in 3′ position polyacylation by transferring the *p*-coumaroyl groups from *p*-coumaroylglucoses to anthocyanins (Noda et al., 2006). *DgSCPL2* of *D. grandiflorum*, belonging to the Clade Ia subfamily, was involved in biosynthesizing 7-polyacylated anthocyanins combined with *DgAA7GT* and *DgAA7BG-GT* (Nishizaki et al., 2013). Thus, we regarded *ScSCPL2* as a key structural gene mainly involved in the 3′ and 7-position acylation for blue flower formation in cineraria.

### *ScMYB2* and *ScMYB4* Might Be Involved in the Transcriptional Regulation of Polyacylation Modification

MYB, bHLH, and WD40 are the major regulatory genes that regulate the expression of structural genes in the anthocyanin biosynthesis pathway. In recent years, many studies have found that MYB transcription factors also regulate the expression of genes specifically related to anthocyanin modification. In *G. triflora*, *GtMYB3* could interact with *GtbHLH1* to activate the expression of *GtA5/3*′*AT* (Nakatsuka et al., 2008). *DcMYB113* was expressed specifically in the roots of purple carrot and was involved in regulating the biosynthesis of anthocyanins by activating the expression of *DcUCGXT1* and *DcSAT1*, which encoded uridine diphosphate glycosyltransferase and SCPL acyltransferase (Xu et al., 2020). *MYBA1* activated the expression of *VvSCPL5*, which was found in *V. vinifera* (Bontpart et al., 2018). This study found that *ScMYB2* and *ScMYB4* were highly expressed in VeB, while almost no expression was detected in VeW and VeC by comparative transcriptomic and gene expression analysis (Figure 9B). According to K-means analysis and gene coexpression network analysis, *ScMYB2*, *ScMYB4* and the key candidate structural genes *ScUGT1*, *ScUGT4*, *ScAGGT11*, *ScAGGT12*, and *ScSCPL2*, which might be involved in the polyacylation modification of cineraria, were found in cluster 8 (Figure 9C). Therefore, we speculated that *ScMYB2* and *ScMYB4* were involved in the transcriptional regulation of polyacylation modification in blue cineraria.

Phylogenetic analysis suggested that *ScMYB2* was clustered into subfamily 4 in the R2R3-MYB transcription factor family (Figure 9A). Previous study found that members in subfamily 4 mainly regulate the biosynthesis of flavonoids in plants. In *Petunia hybrida*, *PhMYB27* was known as a transcription factor regulating the biosynthesis of flavonoids and can inhibit the expression of *PhAN1* and *PhDFR* with an EAR motif in its protein sequence (Albert et al., 2014). *MYB165* and *MYB194* interacted with *bHLH131* to inhibit the transcriptional activation effect of *MYB134* on related genes, such as *ANR*, *DFR*, *LAR*, and *ANS*, in the flavonoid metabolism pathway in *Populus tremula* × *tremuloides* (Ma et al., 2018). Furthermore, members of subfamily 4 have also been found to be directly involved in the regulation of the phenylalanine metabolism pathway. *AtMYB3* in *A. thaliana* combined with the *C4H* promoter directly to inhibit its activation. In the *AtMYB3*-overexpressing lines, the expression of *CHS*, *4CL1*, and *4CL3* decreased significantly, which led to a reduction in the content of malic acid and anthocyanins (Zhou et al., 2017). The above studies showed that members in subfamily 4 of the R2R3-MYB transcription factor family have diverse functions in different plant species. Thus, we hypothesized that *ScMYB2* had the ability to regulate the expression of *ScUGT1*, *ScUGT4*, *ScAGGT11*, *ScAGGT12*, and *ScSCPL2. ScMYB4* belonged to the R3-MYB transcription factor family (Figure 9A), which usually functions as a key repressor in anthocyanin metabolism (Sara et al., 2018). In *Chrysanthemum* × *morifolium*, R3 MYB transcription *CmMYB^#^7* inhibited the biosynthesis of anthocyanins by competing with *CmMYB6* to combine *CmbHLH2* (Xiang et al., 2019). Therefore, we speculated that *ScMYB4* may produce new transcriptional activation or inhibit the activity of other *MYB* inhibitors to induce cineraria accumulation of a large amount of cinerarin. However, the specific function of *ScMYB4* needs further analysis.

## Conclusion

In this study, we found that 3′,7-polyacylated anthocyanin, cinerarin, was the main pigment component that determined the blue colour of ray florets of cineraria by HPLC-MS/MS analysis. Based on comparative transcriptomic analysis combined with RT- and qRT-PCR, we found two genes encoding uridine diphosphate glycosyltransferase, named *ScUGT1* and *ScUGT4*; two genes encoding acyl-glucoside-dependent glucosyltransferases which belong to glycoside hydrolase family 1 (GH1), named *ScAGGT11* and *ScAGGT12*; one gene encoding SCPL acyltransferase *ScSCPL2*; and two MYB transcriptional factor genes *ScMYB2* and *ScMYB4*, that were specifically highly expressed in the ray florets of VeB, which indicated that these genes may be involved in cinerarin biosynthesis. Among them, *ScUGT1* was used to prepare the acyl donor for acylation by caffeic acid, *ScUGT4* may be involved in the glycosylation of 3′-*O*-position. Sc*AGGT*11 and Sc*AGGT*12 were speculated to be involved in the glycosylation of 7-*O*-position. ScSCPL2 mainly participated in the 3′ and 7-position acylation of cinerarin. *ScMYB2* and *ScMYB4* may specifically regulate the expression of these modified genes. Besides, *ScUGT23* and *ScUGT39* that highly expressed in VeC and VeB may be involved in modifying the 3-position glycosylation, which was the most conventional and common glycosylation of anthocyanins but not the key step for blue flower formation. These results will provide new insight into the molecular basis of the polyacylated anthocyanin biosynthesis mechanism in higher plants and are of great significance for blue flower molecular breeding of ornamental plants.

## Data Availability Statement

The RNA-Seq data and Iso-Seq data have been deposited in NCBI under SRA accession codes: PRJNA674976.

## Author Contributions

HH and SD conceived and designed this study. YL, YC, HH, and JR performed the experiments. CL, YL, HH, and JQ carried out the data analysis. CL, HH, YL, and FQ wrote this manuscript. All authors read and approved the final manuscript.

## Conflict of Interest

The authors declare that the research was conducted in the absence of any commercial or financial relationships that could be construed as a potential conflict of interest.

## Footnotes

1 http://www.genome.jp/

2 http://www.r-project.org/

3 http://www.kobas.co.uk/

4 www.cytoscape.org
