# Supplementary material for: Isolation and Functional Analysis of Genes Involved in Polyacylated Anthocyanin Biosynthesis in Blue Senecio cruentus
Source: Front Plant Sci. 2021 Feb 22;12:640746. doi: 10.3389/fpls.2021.640746 (PMC7937962; doi:10.3389/fpls.2021.640746)
Supplement: Supplementary file 1 [file Data_Sheet_1.docx]

**
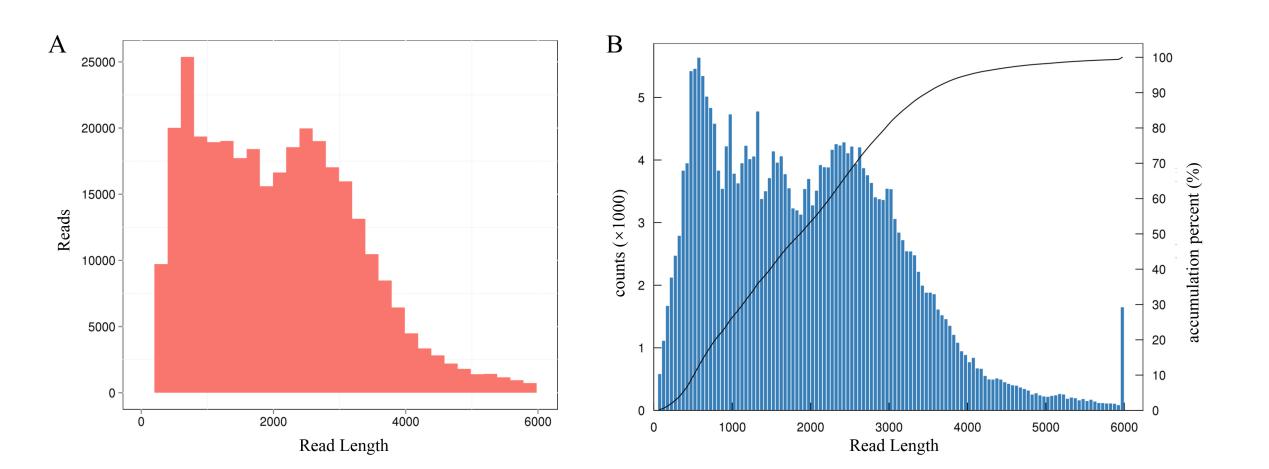
**

**Supplementary Figure 1** Circular consensus sequences (A) and full-length non-chimeric sequences (B) read length distribution of each size bins.

**
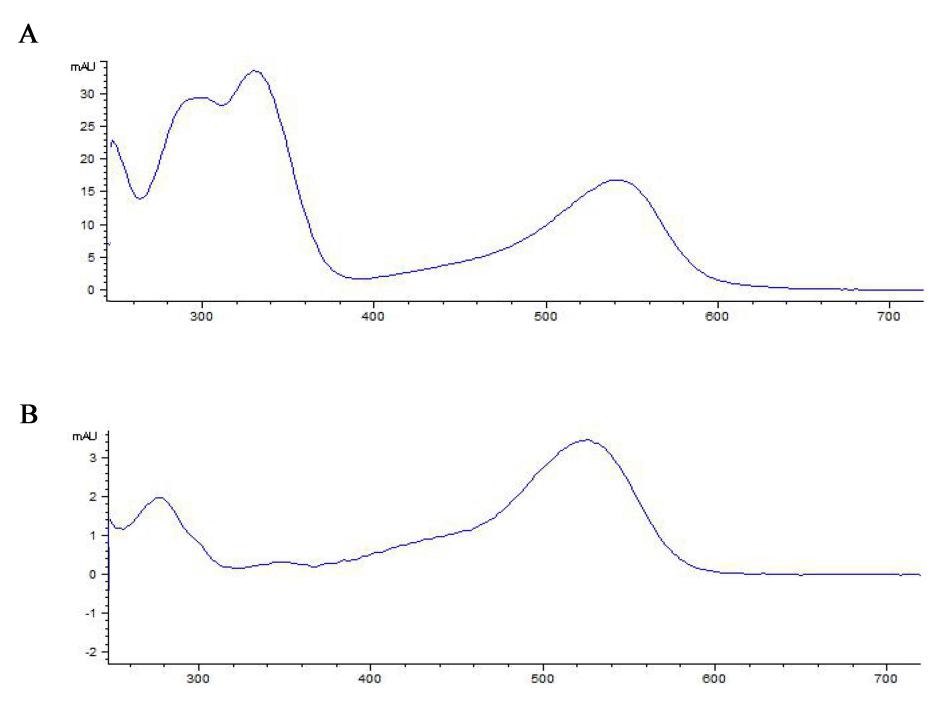
**

**Supplementary Figure 2** UV-vis absorption spectra of B1 peak (A) and B1’ peak (B).

**Supplementary Table 1** Primers used for RT-PCR.

| **Gene name** | **Forward (5**′**-3**′) | **Reverse (5**′**-3**′) |
| --- | --- | --- |
| *ScUGT1* | ATTATTACTTCAACGGCTACGGAG | TTCGGCTTCAACATTGTCCCT |
| *ScUGT3* | CTATTATTACTTCAACGGCTACGA | TCGGCTTCAACATTGTCCCT |
| *ScUGT4* | TTAGAACCGAAAGCACCAGA | CTACAATAGCAACCCCACCA |
| *ScUGT13* | ATACGGGAATGATAGATGTGGC | CGAATACACTGACGGGAAGC |
| *ScUGT14* | AAACCCAACCCAGCACTTAC | GACAAACGAGGAATCCCAAA |
| *ScUGT15* | ATCCAGTCACTTGTATCGTTTGTG | TTCAGCCATTTGAGCCATTT |
| *ScUGT17* | CAGCAGTGACCCAGTTACGC | CCTTTTGTGGTGCCCATTTT |
| *ScUGT19* | CTGTTGGAGGGTTCGTGTCT | CTGCCGCTCTACTCTTGTCA |
| *ScUGT21* | TTCGGGATGGTAAATGCTTC | TAGTATCGCTGGCTGTGCTG |
| *ScUGT22* | CGAGTACAAGGACTTATCCGACC | TGATTAGAAGAAAGGGCACCA |
| *ScUGT23* | AAAGGGTTCTTAGATAAAATAAGTG | AACATCTACAAAACGATCGATTC |
| *ScUGT24* | GAACTCGCCAAACTTATCCA | TTCCTCTAACCCAACAAAACTG |
| *ScUGT25* | GATACCGAGTTAGTGGCTGTCC | CGAACCCATCATCAAACCTATT |
| *ScUGT27* | CGGTTAGTTTGCTTTCAGGG | GCTAGTATCATAACTCCAGCCACA |
| *ScUGT28* | GGGTTTCTGAGTTGGACGCT | CCAGTTGGGCTTTGAATGAT |
| *ScUGT29* | AAGGATTGTCTTGTTCGGTTGT | TCGGAACTGGAAATCTTTGG |
| *ScUGT31* | CCCACTGCACAAAGTAAGCC | CGTCACAATTTCAACTCCCAT |
| *ScUGT32* | ATTTTCGGGTTCCAAGTTTC | CTATCTTCCAGACATCCGTCAC |
| *ScUGT39* | CTTAATGTTGGACCTGAAGATGAAA | CCAATGCAAGTACCTCGTGAG |
| *ScAGGT2* | TTATTATTTTACGGTAGTATTTGGA | TAGTCTTCTATTGATTTTCGACTTA |
| *ScAGGT3* | CATGACTACAGGGGTATACGGG | TACCATGCTGCGTAGAGGTCTAT |
| *ScAGGT5* | AAGTCGAAAATCAATAGAAGACTAT | GCGATCCAACCAACATTGA |
| *ScAGGT9* | ATTTCTTGTTTTTACTATTTTATGG | GAGATGAAAGGAAAGAGATAATGAC |
| *ScAGGT10* | GCCAATCAGGTCTCTCTTATTTATG | AAATCCGAATAAATAACCAAACAGA |
| *ScAGGT11* | CCAATCAGGTTGAGGGAGC | TATGCAACAAAGTCTTCTATTGATT |
| *ScAGGT12* | AATGTTGATTCTTCTAGGTCTTGTT | GATAATCTATAATGTTGCAGACCCA |
| *ScAGGT13* | CCAATCAGGTTGAGGGAGC | GTAAAACACGATCACCGAATTCT |
| *ScAGGT23* | AGCATATCAGACACCTTCGTTC | CTAAGCCATCCGTCATACTCA |
| *ScAGGT31* | GAAGATGGCAGGACATTTAGC | CCAGACAGGGATTGTTCATTAG |
| *ScAGGT38* | GTATGACGGATGGCTTAGTAGAA | CGTATAGAAGTGCTTGGTCTCC |
| *ScSCPL2* | GGTGTAGGAAAAGATGAAGCG | GGAATAACAATCCCCGAATAAG |
| *ScSCPL8* | ATTCCCGTTTGGATTTTCAG | TTCACCTTTTCTTGCCTTCG |
| *ScSCPL18* | GGCGTGTAACGGGAACTATT | GCCACTTTCTCCATTCATCG |
| *Scβ-actin* | TCCACATGCCATTCTTCGTCT | CAAAGCGGTAATTTCCTTGCT |

**Supplementary Table 2** Primers used for qRT-PCR.

| **Gene name** | **Forward (5**′**-3**′) | **Reverse (5**′**-3**′) |
| --- | --- | --- |
| *ScUGT1* | AACCCGACCCTTCTATTCGCGAA | CCAAAATCCGAGACAAACTGTTCCA |
| *ScUGT4* | GATACATCCAACTCCCTAATAAGAG | GGTGGTTATTATGGTTGTTTGT |
| *ScAGGT11* | AATAATGTTGATTCTTCTTCGTCTT | GGTATTAGATGTCTGAACGAAGGTG |
| *ScAGGT12* | TGTTGATTCTTCTAGGTCTTGTTCT | TCCCTCAGCCTGAATGGCA |
| *ScSCPL2* | GCCTAATTGCGACTACACAACTGA | GCGAGAATGATACCCATAGGAGTA |
| *ScMYB1* | CAGAAACTGTCGAGGAGACACA | TAAGTCTTACAATCCCTCCACATC |
| *ScMYB2* | AATAAGGGAGCTTGGTCTAAGG | GAGCATGGAGCCTGATTATGAGA |
| *ScMYB3* | CTAGAAGAAAATTGCTTAATAACATCAT | CGAAACAACCGTCCCACTTCATCA |
| *ScMYB4* | ACTCGAATACTTATGGCTAGTTTGA | ATTTCACGGTCTAACCCATACATCA |
| *Scβ-actin* | GGCTTACATTGCTCTGGACTAT | TGGCTGGAACAACACCTCTG |

**Supplementary Table 3** Primers for the pTRV2::*ScSCPL2* construction.

| **Primer name** | **Sequence（5**′**-3**′**）** | **Amplicon length** |
| --- | --- | --- |
| *vScSCPL2*-F | CGACGACAAGACCCTTCGAATATGCTAAGGAGATGGGA | 368 |
| *vScSCPL2*-R | GAGGAGAAGAGCCCTCTTCAACCTTGGCTTGCGTA |  |

**Supplementary Table 4** Mass spectrometric features of anthocyanins Detected in the leaves and ray florets of VeW and VeB.

| **Components** | **Retention time (min)** | **[M+H]^+^** | **MS/MS (m/z)** |
| --- | --- | --- | --- |
| B1 | 29.1 | 875.1 | 627.1, 551.1, 303.1 |
| B2 | 36.8 | 1199.1 | 1037.2, 875.2, 551.1, 465.2, 303.1, 163.1 |
| B3 | 47.8 | 1523.2 | 1361.2, 1119.2, 875.1, 627.1, 551.1, 303.1, 163.1 |
| B4 | 49.1 | 1507.2 | 1345.2, 1183.2, 859.2, 535.1, 287.1, 163.1 |
| C1 | 41.8 | 859.1 | 611.1, 449.1, 287.1 |
| C2 | 53.5 | 1507.1 | 1345.1, 1183.1, 859.2, 535.1, 287.1 |

**Supplementary Table 5** Statistics of the number of annotated transcripts.

| **Annotated databases** | **Transcript Number** |
| --- | --- |
| COG | 8,639 |
| GO | 14,813 |
| KEGG | 8,972 |
| KOG | 12,485 |
| Pfam | 16,840 |
| SwissProt | 14,669 |
| eggNOG | 18,652 |
| NR | 18,844 |
| all | 18,882 |

**Supplementary Table 6** Mass spectrometric features of anthocyanins Detected in the CK and *ScSCPL2*-silenced tissues.

| **Components** | **Retention time (min)** | **[M+H]^+^** | **MS/MS (m/z)** |
| --- | --- | --- | --- |
| B1’ | 27.9 | 875.1 | 713.1, 627.1, 551.1, 303.1 |
| B2’ | 36.2 | 1199.1 | 1037.2, 875.2, 551.1, 465.2, 303.1, 163.1 |
| B3’ | 47.1 | 1523.2 | 1361.2, 1119.2, 875.1, 627.1, 551.1, 303.1, 163.1 |
| B4’ | 49.0 | 1507.2 | 1345.2, 1183.2, 859.2, 535.1, 287.1, 163.1 |
| B1 | 29.1 | 875.1 | 627.1, 551.1, 303.1 |
| B2 | 36.8 | 1199.1 | 1037.2, 875.2, 551.1, 465.2, 303.1, 163.1 |
| B3 | 47.8 | 1523.2 | 1361.2, 1119.2, 875.1, 627.1, 551.1, 303.1, 163.1 |
| B4 | 49.1 | 1507.2 | 1345.2, 1183.2, 859.2, 535.1, 287.1, 163.1 |
